# Cerenkov Radiation Energy Transfer (CRET) Imaging: A Novel Method for Optical Imaging of PET Isotopes in Biological Systems

**DOI:** 10.1371/journal.pone.0013300

**Published:** 2010-10-11

**Authors:** Robin S. Dothager, Reece J. Goiffon, Erin Jackson, Scott Harpstrite, David Piwnica-Worms

**Affiliations:** 1 BRIGHT Institute, Washington University School of Medicine, St. Louis, Missouri, United States of America; 2 Molecular Imaging Center, Mallinckrodt Institute of Radiology, St. Louis, Missouri, United States of America; 3 Department of Developmental Biology, Washington University School of Medicine, St. Louis, Missouri, United States of America; University of Texas M. D. Anderson Cancer Center, United States of America

## Abstract

**Background:**

Positron emission tomography (PET) allows sensitive, non-invasive analysis of the distribution of radiopharmaceutical tracers labeled with positron (β^+^)-emitting radionuclides in small animals and humans. Upon β^+^ decay, the initial velocity of high-energy β^+^ particles can momentarily exceed the speed of light in tissue, producing Cerenkov radiation that is detectable by optical imaging, but is highly absorbed in living organisms.

**Principal Findings:**

To improve optical imaging of Cerenkov radiation in biological systems, we demonstrate that Cerenkov radiation from decay of the PET isotopes ^64^Cu and ^18^F can be spectrally coupled by energy transfer to high Stokes-shift quantum nanoparticles (Qtracker705) to produce highly red-shifted photonic emissions. Efficient energy transfer was not detected with ^99m^Tc, a predominantly γ-emitting isotope. Similar to bioluminescence resonance energy transfer (BRET) and fluorescence resonance energy transfer (FRET), herein we define the Cerenkov radiation energy transfer (CRET) ratio as the normalized quotient of light detected within a spectral window centered on the fluorophore emission divided by light detected within a spectral window of the Cerenkov radiation emission to quantify imaging signals. Optical images of solutions containing Qtracker705 nanoparticles and [^18^F]FDG showed CRET ratios *in vitro* as high as 8.8±1.1, while images of mice with subcutaneous pseudotumors impregnated with Qtracker705 following intravenous injection of [^18^F]FDG showed CRET ratios *in vivo* as high as 3.5±0.3.

**Conclusions:**

Quantitative CRET imaging may afford a variety of novel optical imaging applications and activation strategies for PET radiopharmaceuticals and other isotopes in biomaterials, tissues and live animals.

## Introduction

Positron emission tomography (PET) allows sensitive, non-invasive measurement of the distribution of picomolar quantities of radiopharmaceuticals labeled with positron (β^+^)-emitting radionuclides (e.g., ^11^C, ^18^F, ^64^Cu, ^68^Ga) in small animals and humans [1]. Upon β^+^ decay, the initial velocity of high-energy β^+^ particles can initially exceed the speed of light in tissue, thereby producing Cerenkov radiation [2], [3]. Unlike fluorescence or emission spectra that have characteristic spectral peaks, Cerenkov radiation spectra are continuous. The relative intensity is proportional to frequency, and thus, for Cerenkov radiation, higher frequencies (ultraviolet/blue) are most intense [2]. Recently, light photons attributed to Cerenkov radiation emitted by common PET isotopes have been detected in live mice *in vivo* using sensitive CCD optical imaging systems [4], [5], [6]. Because the ultraviolet/blue wavelengths of Cerenkov radiation are highly absorbed in living tissues (by water, hemoglobin, cytochromes, etc.), modest signals and heavily surface-weighted images as recorded by external imaging cameras were produced. To overcome these limitations and provide quantitative optical methods to detect β^+^ decay in biological tissues, we hypothesized that Cerenkov radiation could be spectrally coupled by energy transfer to high Stokes-shift quantum nanoparticles (Qtracker705) to produce highly red-shifted emission spectra from the decay of PET isotopes compatible with biological imaging.

## Materials and Methods

### Materials

Athymic nude mice (*nu/nu*; 6 weeks old) were purchased from Taconic (Hudson, NY). [^18^F]FDG and [^64^Cu]CuCl_2_ were obtained from the cyclotron facility, Washington University School of Medicine, St. Louis, MO. [^99m^ Tc]NaTcO_4_ was obtained from the Barnes-Jewish Hospital Nuclear Pharmacy (St. Louis, MO). Qtracker705 quantum dots were purchased from Invitrogen Inc. (Carlsbad, CA). Phenol-free Matrigel was purchased from BD Biosciences (San Jose, CA). Clear-bottom black 96-well plates were purchased from Thermo Fisher Scientific Inc. (Waltham, MA).

### Luminescence Spectra of Cerenkov Radiation and Quantum Dots

Emission spectra were obtained with a spectrophotometer (Cary Eclipse; Varian Inc., Palo Alto, CA) zeroed with 500 µL of PBS in a quartz cuvette. Then, 200 µL of PBS were removed and replaced with 200 µL of [^64^Cu]CuCl_2_ (71.8 MBq; 1.94 mCi) diluted into PBS, and wavelength scanned (20 nm emission slit width, 10 nm interval, 10 second gate, PMT 700 mV, 6 scans). Following baseline scanning, 12.5 µL of a stock solution of Qtracker705 (2 µM) was added to the cuvette (final concentration, 49 nM) and mixed by pipetting. Spectra were obtained as above. This procedure was repeated to achieve spectra for both 222 nM and 400 nM Qtracker705 by addition of appropriate volumes of Qtracker705 stock solution to the cuvette. Each spectrum was the average of 6 scans. Spectra of [^99m^ Tc]NaTcO_4_ alone or in the presence of 400 nM Qtracker 705 were performed identically. The signal at each wavelength in a given scan was decay corrected for time post-addition of activity to the cuvette. After obtaining scans of [^64^Cu]CuCl_2_ with 400 nM Qtracker705, the cuvette was capped, sealed with parafilm, and allowed to decay until no detectable activity remained. Spectra of the decayed sample containing 400 nM Qtracker705 were again obtained. Spectra were also obtained using 4.3 nM of non-radiolabeled CuCl_2_ in PBS alone and in the presence of 400 nM Qtracker705 to serve as non-radioactive controls. A fluorescence emission spectrum of the decayed sample containing 400 nM Qtracker705 was obtained (excitation 350 nm, 10 nm excitation and emission slit widths, 0.5 nm interval, 1 sec averaging, PMT 600 mV). For analysis, data were plotted as RFU at a given wavelength.

### Imaging CRET In Vitro

For imaging the concentration-dependence of Qtracker705 emissions, various aliquots of the 2 µM Qtracker705 stock solution were added to PBS in a black 96-well plate (final concentrations: 0, 10, 25, 50, 100, and 200 nM). [^18^F]FDG diluted in PBS was then added to each well (5.6 MBq (150 µCi) per well at the time of imaging). Each concentration of Qtracker705 was measured in triplicate and the total volume was 100 µL in each well after addition of [^18^F]FDG. Plates were imaged in an IVIS 100 imaging system (Caliper Life Sciences, Hopkinton, MA; binning 8, FOV 15, 1/f stop, 10 sec exposure). Images were captured using open, <510 nm, 500–570 nm, and >590 nm emission filters in rapid succession. For imaging the dose-dependence of [^18^F]FDG, 2 µL of the 2 µM Qtracker705 stock solution was added to PBS in a 96-well plate (final concentration, 40 nM). The volume of [^18^F]FDG stock added to each well was adjusted for radioactive decay (final doses: 0, 0.037, 0.37, 3.7, 37 MBq (0, 1, 10, 100, and 1000 µCi) at the time of imaging). Each dose of radioactivity was measured in triplicate and final total volume (100 µL) was titrated with PBS in each well after addition of [^18^F]FDG. Plates were imaged in the IVIS 100 imaging system (binning 8, FOV 15, 1/f, 30 sec exposure). Images were acquired using open, <510 nm, 500–570 nm, and >590 nm emission filters in rapid succession. To prevent confounding effects from high-energy annihilation photons striking the detector chip in the camera, random outlying pixels (“hot pixels”) were adjusted to the mean value of the nearest neighboring pixels using ImageJ software (National Institutes of Health, Bethesda, MD) prior to analysis. Image analysis and photon flux measurements were determined with Living Image 3.2 and Igor Pro software (Caliper Life Sciences).

### Imaging CRET In Vivo

Animal care and protocols were approved by the Washington University Medical School Animal Studies Committee (Protocol 20090260). To generate imaging phantoms *in vivo*, Matrigel was mixed with either Qtracker705 (200 nM or 500 nM final concentration in PBS) or an equal volume of PBS in a microfuge tube. Athymic nude mice were injected subcutaneously with 150 µL of the Matrigel-Qtracker mix on the right flank or the Matrigel-PBS mix on the left flank, and returned to their cages for 15 minutes while the Matrigel solidified. To collect background images, mice were imaged under 2.5% isoflurane anesthesia in an IVIS 100 imaging system (bining 8, FOV 15, 1/f, 1 min exposure) using open, <510 nm, 500–570 nm, and >590 nm emission filters in rapid succession. Mice were then injected with 100 µL of [^18^F]FDG (17.6 MBq; 475 µCi) in PBS via tail vein and imaged 5 minutes later as above. Mice were reimaged after 30 minutes, which allowed renal clearance of [^18^F]FDG. Hot pixels that occurred over regions of interest that contained pseudotumor phantoms were adjusted to the mean value of the nearest neighboring pixels using ImageJ software prior to further analysis. Photon flux measurements and image analysis were performed using Living Image 3.2 and Igor Pro software (Caliper Life Sciences). Matched red-filter images were divided by blue-filter images, and ROIs over the pseudotumors were used to calculate tumor CRET ratios. For selected images, the mean red/blue pixel values from a ROI over the PBS pseudotumor were subtracted from all pixels to approximate a calculated CRET image.

### Calculation of CRET Ratios

Empirically, in the presence of a fluorophore and Cerenkov radiation, CRET can be calculated in a manner similar to BRET [7], [8] and FRET [9] as the quotient of light detected within a spectral window (*X*) centered on the fluorophore emission divided by light detected within a spectral window (*Y*) of the Cerenkov radiation emission, minus the quotient of light detected in windows *X* and *Y* in the presence of Cerenkov radiation alone (Eq. 1):
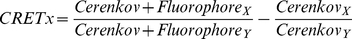
(1)In the case of Qtracker705, then:
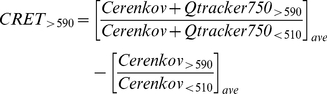
(2)


### Statistical Analyses

Data were reported as mean values ± standard error of the mean (SEM) for the number of wells or animals as indicated in figure legends. Pairs were compared with the Student *t*-test [10] and values of p≤0.05 were considered significant.

## Results

**Figure 1:**
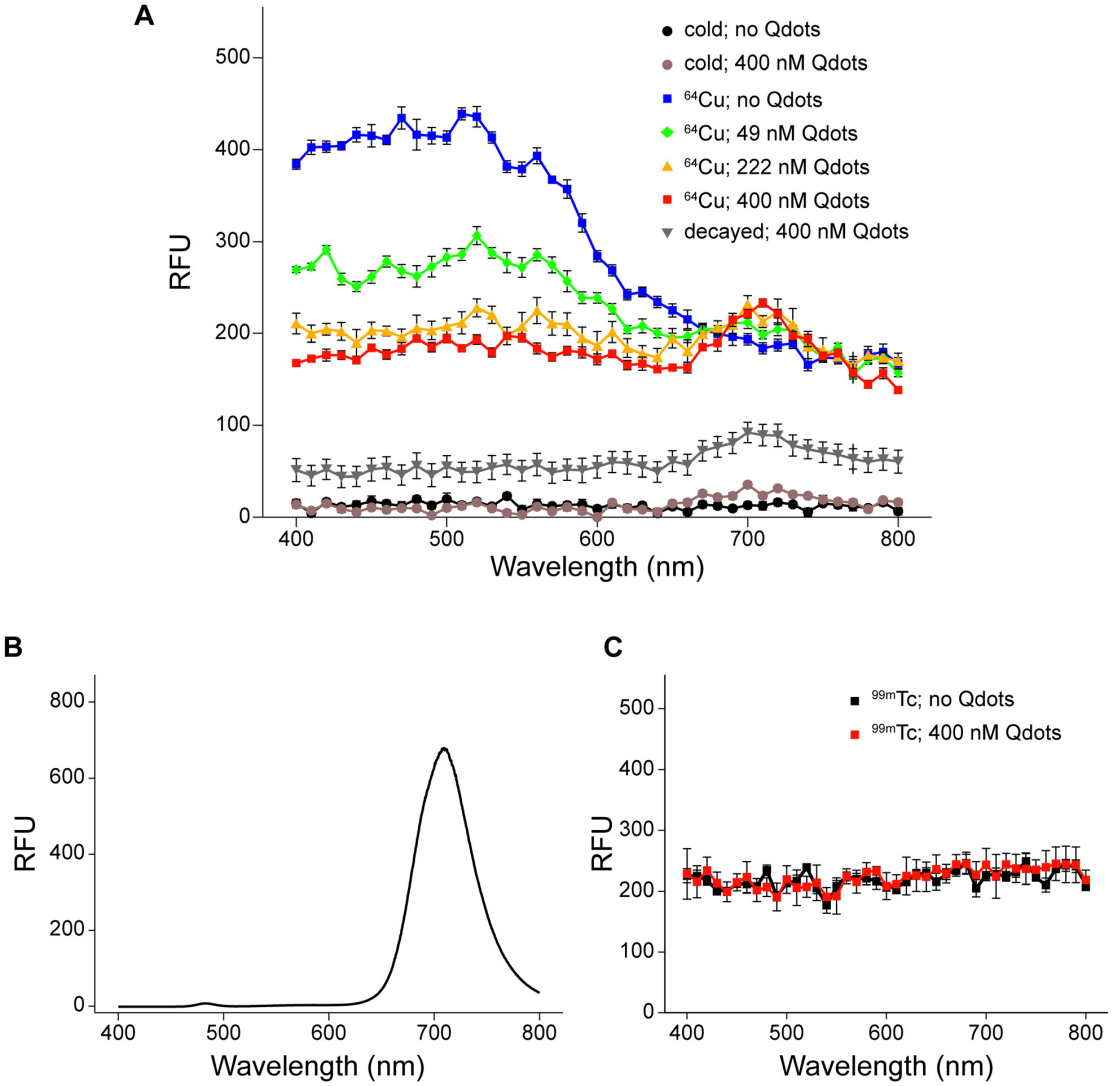
Spectral analysis. (**A**) UV/vis emission spectra of [^64^Cu]CuCl_2_ in PBS containing various concentrations of Qtracker705 nanoparticles (Qdots ) demonstrate Cerenkov radiation energy transfer (CRET); (blue) [^64^Cu]CuCl_2_ without Qdots, (green) [^64^Cu]CuCl_2_ with 49 nM Qdots, (orange) [^64^Cu]CuCl_2_ with 222 nM Qdots, (red) [^64^Cu]CuCl_2_ with 400 nM Qdots, (black) non-radioactive CuCl_2_ without Qdots, (brown) non-radioactive CuCl_2_ with 400 nM Qdots, (gray) decayed [^64^Cu]CuCl_2_ with 400 nM Qdots. (**B**) Fluorescence emission spectrum (350 nm excitation) of decayed (>8 half-lives) [^64^Cu]CuCl_2_ in PBS containing 400 nM Qtracker705. (**C**) UV/vis emission spectra of [^99m^Tc]NaTcO_4_ in PBS without (black) and with (red) 400 nM Qtracker705 nanoparticles.

As a first demonstration, UV/vis emission spectra recorded from phosphate-buffered saline (PBS) containing 70 MBq (1.9 mCi) [^64^Cu]CuCl_2_ (^64^Cu: half-life, 12.7 hrs; β^+^ decay, 19%, 0.655 MeV; β^−^ decay, 39%, 0.578 MeV) showed a broad maximum at 400–550 nm with a monotonic decline out to 800 nm (**Figure 1A**), a spectral shape attributed to the inverse dependence of Cerenkov radiation intensity on wavelength, modified by the wavelength-dependence of the refractive index of water, the depth-dependence of measured spectra, and detector characteristics [2], [11], [12]. [^64^Cu]CuCl_2_ was selected for this experiment because of its long half-life, allowing little decay over the time needed for data collection. Addition of Qtracker705 nanoparticles, selected because their absorption spectra overlap the UV/blue emissions of Cerenkov radiation [13], reduced substantially the 400–550 nm broad band emissions while producing a peak centered on 705 nm. Importantly, the new peak was Qtracker705 concentration-dependent (49 nM to 400 nM) and corresponded to the peak emission of Qtracker705, consistent with Cerenkov radiation energy transfer (CRET). Furthermore, Qtracker705 solution (400 nM) scanned after radioactive decay produced near background emissions. The 705 nm peak was not due to spontaneous non-radioactive emission processes, such as phosphorescence, since equivalent solutions of Qtracker705 (400 nM) in PBS with or without non-radioactive CuCl_2_ (4.3 nM in PBS) showed only background emissions. Qtracker705 nanoparticles were stable; after complete radioactive decay, control *fluorescence* emission spectra of the nanoparticles were unchanged (350 nm excitation; 705 nm peak emission) (**Figure 1B**). The peak centered on 705 nm could not be attributed to γ radiation at these concentrations since Qtracker705 solution (400 nM) spiked with [^99m^Tc]NaTcO_4_ (^99m^Tc: half-life, 6.0 hrs; γ decay, 89%, 0.140 MeV), a predominantly γ-emitting isotope, yielded a flat emission spectra superimposable on PBS alone spiked with [^99m^Tc]NaTcO_4_ (**Figure 1C**).

**Figure 2:**
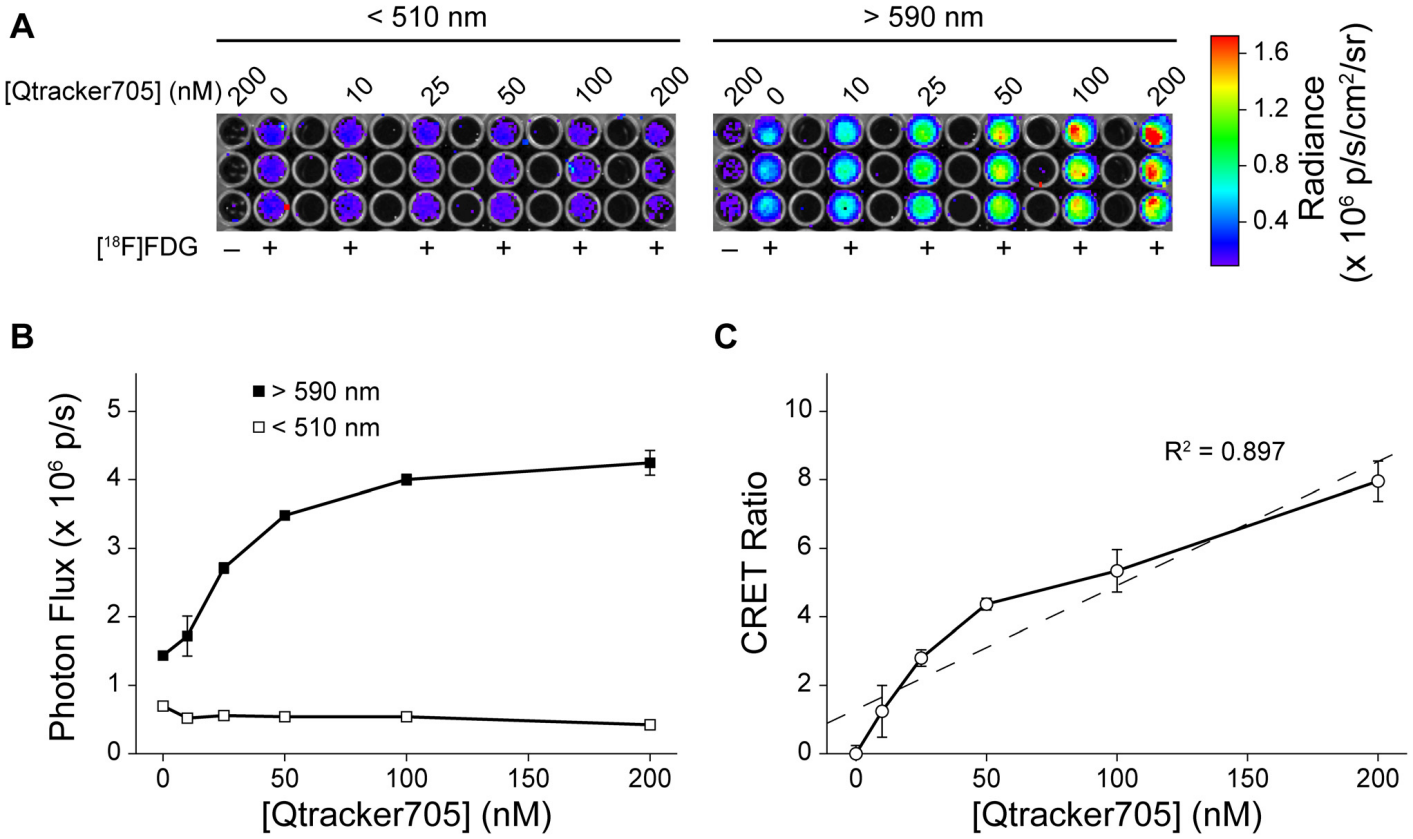
CRET *in vitro* was dependent on Qtracker705 nanoparticle concentration. (**A**) IVIS 100 images of 96-well assay plates using either a <510 nm filter (left) or a >590 nm filter (right). Note the red “hot pixel” from an annihilation event detected by the CCD camera in one image (left), and the presence of minimally detectable CRET emitted from the wells containing 200 nM Qtracker705, but no [^18^F]FDG, due to contaminating radioactive emissions from adjacent wells (right). Qtracker705 nanoparticles show no CRET when imaged in isolation in the absence of [^18^F]FDG. (**B**) Plot of photon flux from either the <510 nm filter (□) or the >590 filter (▪) with increasing concentrations of Qtracker705 nanoparticles. (**C**) Plot of CRET ratios versus concentration of Qtracker705 nanoparticles (dashed line is a linear fit of the data: y = 0.036x+1.3; R^2^ = 0.897).

**Figure 3:**
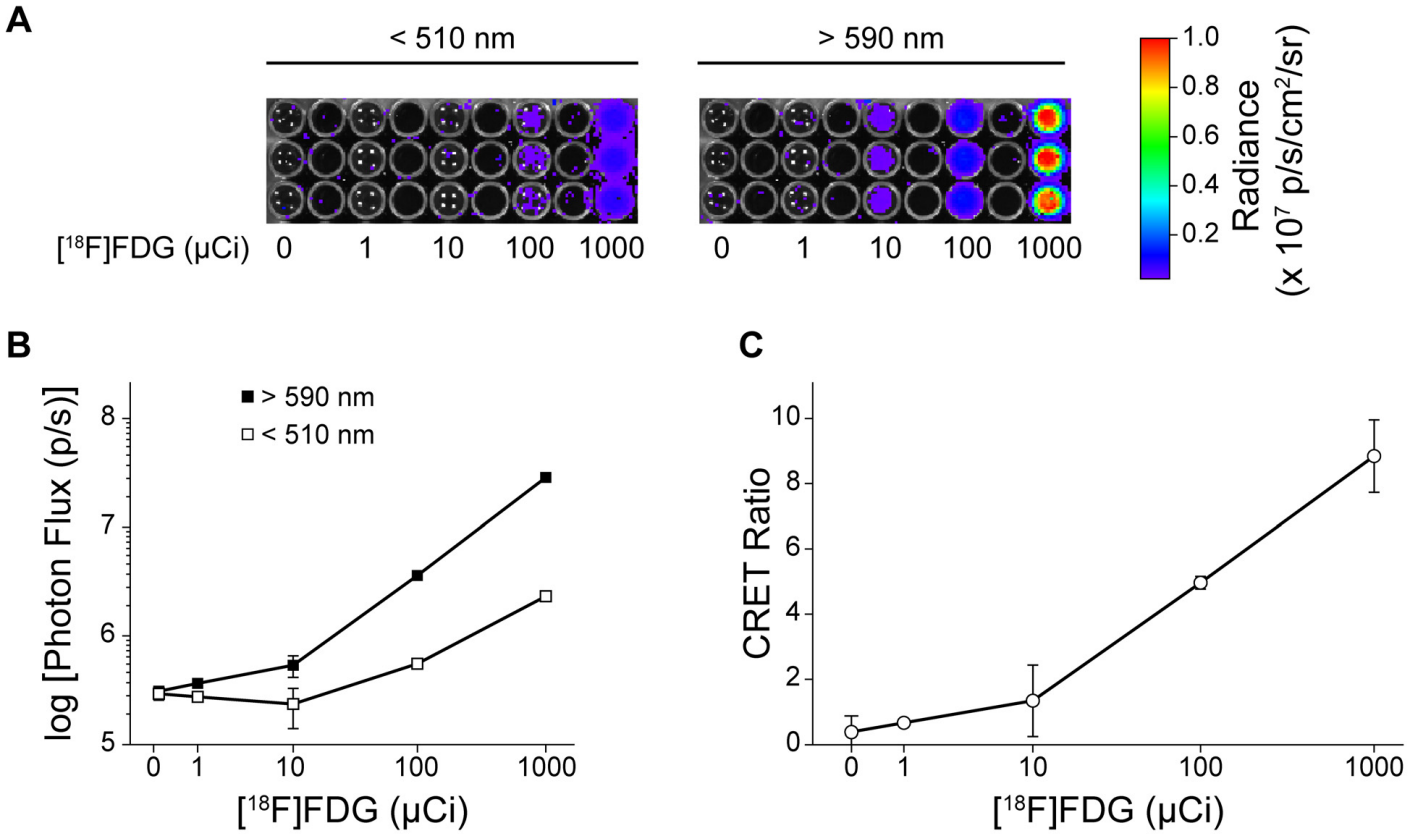
CRET *in vitro* was dependent on [^18^F]FDG radioactivity. (**A**) IVIS 100 images of 96-well assay plates using either a <510 nm filter (left) or a >590 nm filter (right). (**B**) Plot of photon flux from either the <510 nm filter (□) or the >590 filter (▪) with increasing amounts of [^18^F]FDG radioactivity. (**C**) Plot of CRET ratios versus [^18^F]FDG radioactivity.

Using 96-well plates, we then imaged Qtracker705 nanoparticles mixed at several different concentrations in PBS with 5.6 MBq (150 µCi) of [^18^F]fluorodeoxyglucose (FDG) (^18^F: half-life, 1.83 hrs; β^+^ decay, 97%, 0.635 MeV). Images were captured with an IVIS 100 system using open, <510 nm, 500–570 nm, and >590 nm filters in rapid succession to distinguish the bulk of the Cerenkov radiation (<510 nm) from radiation energy transfer to the Qtracker705 nanoparticles (>590 nm) (**Figure 2A**). The photon flux in the red filtered images (>590 nm) increased with increasing concentration of Qtracker705, while the blue filtered images (<510 nm) decreased slightly, consistent with absorption of Cerenkov radiation (**Figure 2B**). We propose use of equation **Eq. 1** (Methods) to quantify CRET by calculation of a CRET ratio. As applied herein, CRET ratios were calculated using **Eq. 2** and, at constant [^18^F]FDG, found to correlate with the concentration of Qtracker705 (**Figure 2C**). Similarly, at constant Qtracker705 concentration (40 nM), CRET ratios correlated with the amount of [^18^F]FDG radioactivity added to the solution (**Figure 3**). Obscuring the plate with a single sheet of black paper completely blocked CRET signals, confirming that the events detected in the IVIS 100 were visible light photons, not high-energy radioactivity (data not shown). CRET ratios *in vitro* as high as 8.1±1.1 (n = 3) were observed.

**Figure 4:**
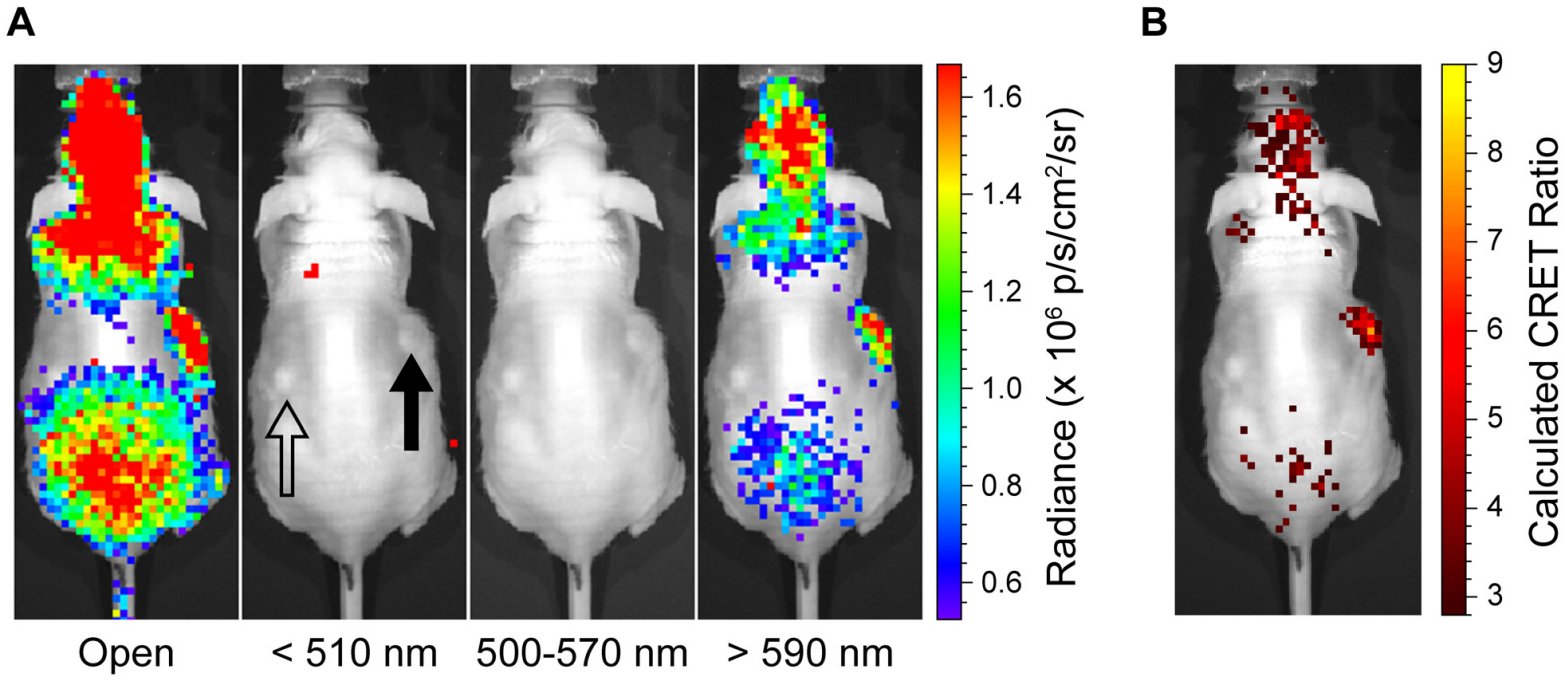
CRET imaging of pseudotumor phantoms in live animals *in vivo*. (**A**) Subcutaneous pseudotumors of 500 nM Qtracker705-impregnated Matrigel (closed arrow) and PBS-impregnated Matrigel (open arrow) in opposing flanks of *nu/nu* mice were imaged with an IVIS 100 using open, <510 nm (blue), 500–570 nm (green), and >590 nm (red) filters 30 minutes following tail-vein injection of [^18^F]FDG (17.6 MBq; 475 µCi). (**B**) The calculated CRET image.

Because red-shifted emissions penetrate biological tissues more readily than blue emissions [14], CRET may enhance optical imaging of PET radiopharmaceuticals in living animals. As proof-of-principle, we embedded Qtracker705 nanoparticles or vehicle (PBS) alone in Matrigel solutions and established identical subcutaneous implants on opposing flanks of nude (*nu/nu*) mice [15] for live animal imaging (**Figure 4A**). We embedded two different concentrations of Qtracker705 (200 nM and 500 nM) in two different cohorts of mice and imaged at 5 minutes and 30 minutes following tail-vein injection of [^18^F]FDG (17.6 MBq; 475 µCi). Five minutes post [^18^F]FDG injection, red/blue image ratios (signal in the >590 nm filtered (red) images divided by the <510 nm (blue) images) for 200 nM Qtracker705-impregnated Matrigel pseudotumors were significantly higher than PBS-containing pseudotumors (5.1±0.2 (n = 4) versus 3.1±0.2, respectively; p<0.0001). The corresponding CRET ratio was 2.1±0.2 *in vivo* (**Table 1**). Thirty minutes post-injection, Qtracker705-containing pseudotumors had red/blue ratios of 4.8±0.1 and PBS-containing pseudotumors had red/blue ratios of 3.0±0.2, yielding a CRET ratio of 1.8±0.2 (**Table 1** **;** **Figure 4B**). By comparison, 5 minutes post [^18^F]FDG injection, 500 nM Qtracker705-impregnated Matrigel pseudotumors had red/blue ratios of 5.5±0.4 (n = 3) and PBS-containing pseudotumors had ratios of 2.0±0.6, corresponding to a CRET ratio of 3.5±0.3 (**Table 1**). Thirty minutes post-injection, 500 nM Qtracker705-containing pseudotumors had red/blue ratios of 4.8±0.6 and PBS-containing pseudotumors had a ratio of 2.6±0.3, yielding a CRET ratio of 2.2±0.3. Cerenkov radiation spectra are continuous, and low level emissions were evident into the far red spectral tail (**Figure 1A**). Thus, areas of known high focal uptake and excretion of [^18^F]FDG (brain, heart, bladder) showed evidence of low level Cerenkov radiation visible with the >590 nm filter (**Figure 4A** **, >590 filtered image**).

**Table 1:** CRET ratios of pseudotumors *in vivo*.

[Qtracker705]	Time Post [^18^F]FDG injection	
	*5 min*	*30 min*
*200 nM*	2.1±0.2	1.8±0.2
*500 nM*	3.5±0.3	2.2±0.3

## Discussion

We describe a method for enhancing optical detection of PET isotopes in biological systems, termed Cerenkov radiation energy transfer (CRET). Similar in principle to BRET [7] and FRET [9], Cerenkov radiation generated by decay of a PET isotope serves as the energy donor and a fluorophore constitutes the energy acceptor. We propose that, in the presence of a fluorophore and Cerenkov radiation, CRET ratios can be calculated as the ratio of light detected within a spectral window centered on the fluorophore emission over light detected within a spectral window of the Cerenkov radiation emission, minus the ratio of light detected in the same filters in the presence of Cerenkov radiation alone (**Eq. 1**). As proof of principle, we quantified CRET ratios by imaging the energy transfer of Cerenkov radiation generated from PET isotopes to Qtracker705 nanoparticles both *in vitro* and *in vivo*.

Spectra obtained with Qtracker705 in the presence of ^64^Cu showed an apparent maximum for the energy donor from 400–550 nm for Cerenkov radiation (attributed to inverse wavelength-, refractive index-, depth- and detector-dependent characteristics) and a new acceptor peak centered on 705 nm corresponding to the emission of Qtracker705, consistent with Cerenkov radiation energy transfer. Additionally, we observed with increasing concentrations of Qtracker705 a concomitant decrease in the donor intensity. Such a loss, concordant with formation of the acceptor spectral peak, is similarly observed in FRET. Furthermore, Cerenkov radiation arises from high-energy particles traveling through a medium, inducing transient dipole-moments, while the mechanism of non-radiative (resonance) energy transfer with FRET involves dipole-dipole coupling. However, attributing resonance processes to the CRET phenomena awaits specific mechanistic studies aimed at understanding all possible energy contributions made by decaying PET isotopes in such energy transfer contexts.

Studies *in vitro* were performed by determining the concentration-dependence of Qtracker705 in the presence of a constant amount of [^18^F]FDG. We chose nanoparticle concentrations in the nanomolar range, comparable to concentrations that would be feasible *in vivo*. Within the narrow concentration range of these experiments, we found a quasi-linear increase in CRET with increasing concentrations of Qtracker705. This trend was expected given the sub-optimal excitation that occurs when using such a low-level light source compared to the high molar absorptivity of quantum dot nanoparticles. Additionally, we examined the dose-dependence of [^18^F]FDG in the presence of a constant amount of Qtracker705. The doses of radiation used in this experiment also were chosen in consideration of applications *in vivo*, but were limited by high-energy annihilation photons interfering with the CCD detector. We found that CRET ratios increased with radiation dose over the range of doses examined.

Note that the CRET ratio will depend quantitatively on the choice of filters, isotope and fluorophore. Further refinement of CRET imaging is anticipated as optimal filter combinations for various isotopes and fluorophores are discovered and characterized. In this regard, the data in Figure 1A suggest the presence of an isosbestic point at ∼680 nm in the CRET spectra of Qtracker705 nanoparticles. Use of narrow bandpass filter sets that include isosbestic points may also enhance quantitative analysis, system calibration, and depth resolution in a variety of experimental conditions that will be explored in the future.

We have demonstrated the imaging applicability of CRET *in vivo* using Matrigel pseudotumor phantoms embedded with Qtracker705 or PBS. Following tail-vein injection of [^18^F]FDG, we were able to correctly observe the location of Qtracker-loaded pseudotumors as early as five minutes post-injection, a time when [^18^F]FDG is widely distributed throughout the blood pool and extracellular spaces. The mean range of positrons emitted by ^18^F and ^64^Cu is ∼0.9 mm (and for very high-energy positron emitters such as ^82^Rb is ∼7 mm) [16]. Thus, [^18^F]FDG, in this case the ultimate source of Cerenkov radiation, did not need to be in direct proximity with the nanoparticles to provide adequate energy for CRET, a potential advantage for CRET over FRET or BRET for selected applications. For this reason, there was no need for vessel growth within the pseudotumor for visualization by CRET. Imaging again at 30 minutes post-injection of [^18^F]FDG resulted in reduced signal, as would be expected as [^18^F]FDG cleared from the blood pool and surface tissues. Conversely, for nanoparticles contained within a vascularized tumor, retention of ^18^F]FDG within the tumor cells would provide enhanced proximity and delayed clearance compared to an extracellular source as illustrated in this study. Indeed, the relationship between distance and signal could be quite complex and will need further characterization as CRET imaging is refined. The experiment also was performed using either 200 nM or 500 nM Qtracker705 and, appropriately, the higher concentration yielded a higher CRET ratio, but did not track linearly with the concentration of Qtracker705 *in vivo*, as was observed *in vitro*. This was likely because of non-linear tissue attenuation of photons in living animals.

As this manuscript was submitted, a report demonstrating the use of low-energy light from γ-emitting ^131^I to excite quantum dot nanoparticles in Matrigel phantoms was published independently [17], but with some notable differences. First, in Liu et al. [17], radiotracer was directly admixed into the Matrigel phantoms, rather than injected systemically as in the present study, thereby nominally concentrating the activity by ∼3,000-fold (volume of distribution: 2×2×2 mm^3^ pseudotumor = 8 mm^3^ versus a 25 gm mouse = 25×10^3^ mm^3^). This favorably allowed use of less radiotracer and quantum nanoparticle material, while reducing background signal, but did not mimic the manner in which this technique would likely be utilized *in vivo*. Additionally, ^131^I was chosen as the energy donor, which produced emissions dominated by both γ-rays and β^−^ particles, whereas use of ^18^F in the present study, a nearly pure positron emitter, formally confirmed β^+^ particles as the energy donors and left little ambiguity as to the source of the donor Cerenkov radiation observed. Last, herein we introduce a method for quantitative analysis of the energy transfer process by defining the CRET ratio.

Several optical imaging studies report using Cerenkov radiation as a means of imaging tumors [4], [6], [17], including a recent description of Cerenkov luminescence tomography [18]. While the authors were able to correctly identify tumors using this method, by comparison to traditional PET imaging, Cerenkov luminescence imaging alone resulted in substantially lower spatial resolution. This was likely a consequence of the models used for image reconstruction and photonic limitations. Such reconstructions depend on the ability to accurately model photon propagation through heterogeneous tissues. The UV/blue emissions of Cerenkov radiation are highly absorbed in tissues, resulting in relatively short mean pathlengths for these purposes. Thus, one way to overcome the problem of optical diffusion may be to spectrally couple, by energy transfer, the Cerenkov radiation to far-red and near infrared emitting quantum nanoparticles or fluorophores.

While we focus on Cerenkov radiation from β^+^ particles as the source of excitation energy in this report, in principle, the method should apply to any isotope that emits charged particles that exceed the energy threshold required for Cerenkov radiation in the media (264 keV in water). Thus, β^−^ particles and α-particles of sufficient energy [6] should also enable CRET. It is also possible that other sources of high-energy radiation, such as Bremsstrahlung, radioluminescence from γ-rays, and non-radiative (resonance) energy transfer may also enable energy transfer and the detection of “CRET-like” images. As such, further mechanistic and chemical studies are warranted with other fluorophores and small molecules to determine their relative contributions, strength of signal, and tissue depth-dependence. Quantitative CRET imaging may afford a variety of novel optical imaging applications and activation strategies for studies of PET and other radiopharmaceuticals as well as radiobiology in biomaterials, tissues and live animals.

## References

[1] Ametamey SM, Honer M, Schubiger PA (2008). Molecular Imaging with PET.. Chem Rev.

[2] Jelley JV (1955). Cerenkov radiation and its applications.. Br J Appl Phys.

[3] Ross HH (1969). Measurement of β-emitting nuclides using Cerenkov radiation.. Anal Chem.

[4] Robertson R, Germanos M, Li C, Mitchell G, Cherry S (2009). Optical imaging of Cerenkov light generation from positron-emitting radiotracers.. Phys Med Biol.

[5] Spinelli A, D'Ambrosio D, Calderan L, Marengo M, Sbarbati A (2010). Cerenkov radiation allows in vivo optical imaging of positron emitting radiotracers.. Phys Med Biol.

[6] Ruggiero A, Holland J, Lewis J, Grimm J (2010). Cerenkov luminescence imaging of medical isotopes.. J Nucl Med.

[7] Gammon S, Villalobos V, Roshal M, Samrakandi M, Piwnica-Worms D (2009). Rational design of novel red-shifted BRET pairs: platforms for real-time single chain protease biosensors.. Biotechnol Prog.

[8] Xu Y, Piston DW, Johnson CH (1999). A bioluminescence resonance energy transfer (BRET) system: application to interacting circadian clock proteins.. Proc Natl Acad Sci U S A.

[9] Jares-Erijman EA, Jovin TM (2003). FRET imaging.. Nat Biotechnol.

[10] Glantz SA (1987).

[11] Lambert J, Yin Y, McKenzie DR, Law S, Suchowerska N (2009). Cerenkov light spectrum in an optical fiber exposed to a photon or electron radiation therapy beam.. Appl Opt.

[12] Cho J, Taschereau R, Olma S, Liu K, Chen Y-C (2009). Cerenkov radiation imaging as a method for quantitative measurements of beta particles in a microfluidic chip.. Phys Med Biol.

[13] Bruchez M, Moronne M, Gin P, Weiss S, Alivisatos AP (1998). Semiconductor nanocrystals as fluorescent biological labels.. Science.

[14] Gammon ST, Leevy WM, Gross S, Gokel GW, Piwnica-Worms D (2006). Spectral unmixing of multicolored bioluminescence emitted from heterogeneous biological sources.. Anal Chem.

[15] Gross S, Gammon S, Moss BL, Rauch D, Harding J (2009). Bioluminescence imaging of myeloperoxidase activity in vivo.. Nature Medicine.

[16] Tai Y, Laforest R (2005). Instrumentation aspects of animal PET.. Ann Rev Biomed Eng.

[17] Liu H, Zhang X, Xing B, Han P, Gambhir S (2010). Radiation-luminescence-excited quantum dots for in vivo multiplexed optical imaging.. Small.

[18] Li C, Mitchell G, Cherry S (2010). Cerenkov luminescence tomography for small-animal imaging.. Opt Lett.

